# Dabigatran ameliorates airway smooth muscle remodeling in asthma by modulating Yes‐associated protein

**DOI:** 10.1111/jcmm.15485

**Published:** 2020-06-15

**Authors:** Zhenan Deng, Haojun Xie, Weiying Cheng, Meihong Zhang, Jie Liu, Yating Huo, Yulin Liao, Yuanxiong Cheng

**Affiliations:** ^1^ Department of Respiratory and Critical Care Medicine The Third Affiliated Hospital Southern Medical University Guangzhou China; ^2^ Department of Cardiology Nanfang Hospital Southern Medical University Guangzhou China

**Keywords:** airway remodeling, airway smooth muscle, asthma, dabigatran, thrombin, Yes‐associated protein

## Abstract

Accumulating evidence indicates that thrombin, the major effector of the coagulation cascade, plays an important role in the pathogenesis of asthma. Interestingly, dabigatran, a drug used in clinical anticoagulation, directly inhibits thrombin activity. The aim of this study was to investigate the effects and mechanisms of dabigatran on airway smooth muscle remodeling in vivo and in vitro. Here, we found that dabigatran attenuated inflammatory pathology, mucus production, and collagen deposition in the lungs of asthmatic mice. Additionally, dabigatran suppressed Yes‐associated protein (YAP) activation in airway smooth muscle of asthmatic mice. In human airway smooth muscle cells (HASMCs), dabigatran not only alleviated thrombin‐induced proliferation, migration and up‐regulation of collagen I, α‐SMA, CTGF and cyclin D1, but also inhibited thrombin‐induced YAP activation, while YAP activation mediated thrombin‐induced HASMCs remodeling. Mechanistically, thrombin promoted actin stress fibre polymerization through the PAR1/RhoA/ROCK/MLC2 axis to activate YAP and then interacted with SMAD2 in the nucleus to induce downstream target genes, ultimately aggravating HASMCs remodeling. Our study provides experimental evidence that dabigatran ameliorates airway smooth muscle remodeling in asthma by inhibiting YAP signalling, and dabigatran may have therapeutic potential for the treatment of asthma.

## 1 INTRODUCTION

Asthma is a common non‐infectious chronic disease that seriously endangers human health, affecting nearly 334 million people worldwide,^1^ and its main features are chronic airway inflammation, airway hyper‐responsiveness and airway remodeling.^2^, ^3^ Airway remodeling can be defined as airway wall thickening due to repeated structural changes in the airways caused by repeated airway wall damage and repair, the severity of which is closely related to the progressive decline in lung function of asthmatic patients.^4^ The thickening of airway smooth muscle layer seems to be the most important feature of airway remodeling, mainly characterized by abnormal proliferation, migration and excessive extracellular matrix (ECM) and muscle protein expression of airway smooth muscle cells.^5^

Recent years, several studies showed that the coagulation system was activated in the asthmatic airway, suggesting that it may be related to the pathogenesis of asthma.^6^, ^7^ Thrombin, the major effector of the coagulation cascade, was detected in elevated level of induced sputum and bronchoalveolar lavage fluid (BALF) in asthmatic patients.^8^, ^9^ In vitro, thrombin augmented inflammation of airway cells via cleavage of protease‐activated receptors ^10^ and stimulated contraction of human bronchial rings.^11^ In addition, thrombin is also a potent inducer of cell proliferation, pro‐inflammatory chemokines and ECM proteins of lung fibroblasts.^12^ Our previous study reported that thrombin promoted HASMCs proliferation, and for the first time, thrombin was found to stimulate the production of ECM proteins in HASMCs.^13^ Dabigatran, a novel oral thrombin inhibitor for clinical anticoagulation, was shown to exert anti‐inflammatory and anti‐fibrotic effects in a mouse model of bleomycin‐induced pulmonary fibrosis,^14^ and dabigatran also impaired thrombin‐induced cell proliferation, α‐SMA expression and organization, and production of collagen and connective tissue growth factor (CTGF) in lung fibroblasts.^15^ However, the effects of dabigatran on airway remodeling in OVA‐evoked asthmatic mice and on thrombin‐induced HASMCs remodeling have not been reported.

Yes‐associated protein, a transcriptional coregulator, is a key effector of the Hippo pathway that regulates organ size and tissue homeostasis by affecting cell proliferation and differentiation.^16^ When the Hippo pathway is OFF, YAP is dephosphorylated and accumulates in the nucleus, where they bind to corresponding transcription factors to induce the transcription of downstream target genes such as CTGF and cyclin D1.^17^ Recent studies indicated that YAP was up‐regulated in the bronchial airway smooth muscle of OVA‐evoked asthmatic mice, suggesting that YAP could be associated with airway smooth muscle remodeling in asthma.^18^ It was shown that S1P stimulated proliferation, migration and contraction of rat airway smooth muscle cell by activating YAP,^19^ and Jung‐Soon Mo et al^20^ found that TRAP6, a selective PAR1‐activating peptide, stimulated MDA‐MB‐231 cell migration and invasion by modulating YAP dephosphorylation. Interestingly, the biological role of thrombin is mainly transduced by proteolytic cleavage of protease‐activated receptors (PARs), a family of G‐protein‐coupled receptors (GPCRs) composed of four members (PAR1‐PAR4), of which PAR1, PAR3 and PAR4 are activated by thrombin, and PAR2 is activated by coagulation factors FXa, FVIIa and other proteases.^21^ In addition, We previously confirmed that thrombin induced actin stress fibre polymerization in HASMCs,^13^ while changes in actin cytoskeleton represent a central mechanism for controlling YAP activity.^22^, ^23^ However, the role of YAP in asthma, whether thrombin activates YAP in HASMCs and its underlying mechanisms remain unclear.

Based on these findings, this study aimed to characterize the role of dabigatran in airway smooth muscle remodeling in asthma and explore the underlying mechanisms.

## 2 MATERIALS AND METHODS

### 2.1 Chemicals and reagents

HE, PAS and Masson's trichrome staining kits were purchased from Nanjing Jiancheng Biology Engineering Institute (Nanjing, Jiangsu, China). Dulbecco's modified Eagle's medium (DMEM), foetal bovine serum (FBS), penicillin and streptomycin were purchased from GIBCO BRL. Thrombin and dexamethasone powder were from Sigma‐Aldrich Co. Dabigatran was obtained from Boehringer Ingelheim Pharma. Inhibitors used were PAR1 antagonist SCH79797 (Tocris Bioscience), RhoA inhibitor Y‐16, ROCK inhibitor Y‐27632, YAP inhibitor verteporfin (MedChemExpress) and the actin polymerization inhibitor Cytochalasin D (Abcam). The antibodies used were anti‐collagen I, anti‐CTGF, anti‐β‐actin, anti‐GAPDH (Biosynthesis Biotechnology); anti‐RhoA, anti‐ROCK, anti‐phosphorylated MLC2, anti‐MLC2, anti‐phosphorylated SMAD2, anti‐SMAD2, anti‐phosphorylated YAP, anti‐α‐SMA, anti‐cyclin D1 (Cell Signaling Technology) and anti‐YAP (Santa Cruz Biotechnology).

### 2.2 Establishment of OVA‐evoked asthma mouse model

**Figure 1:**
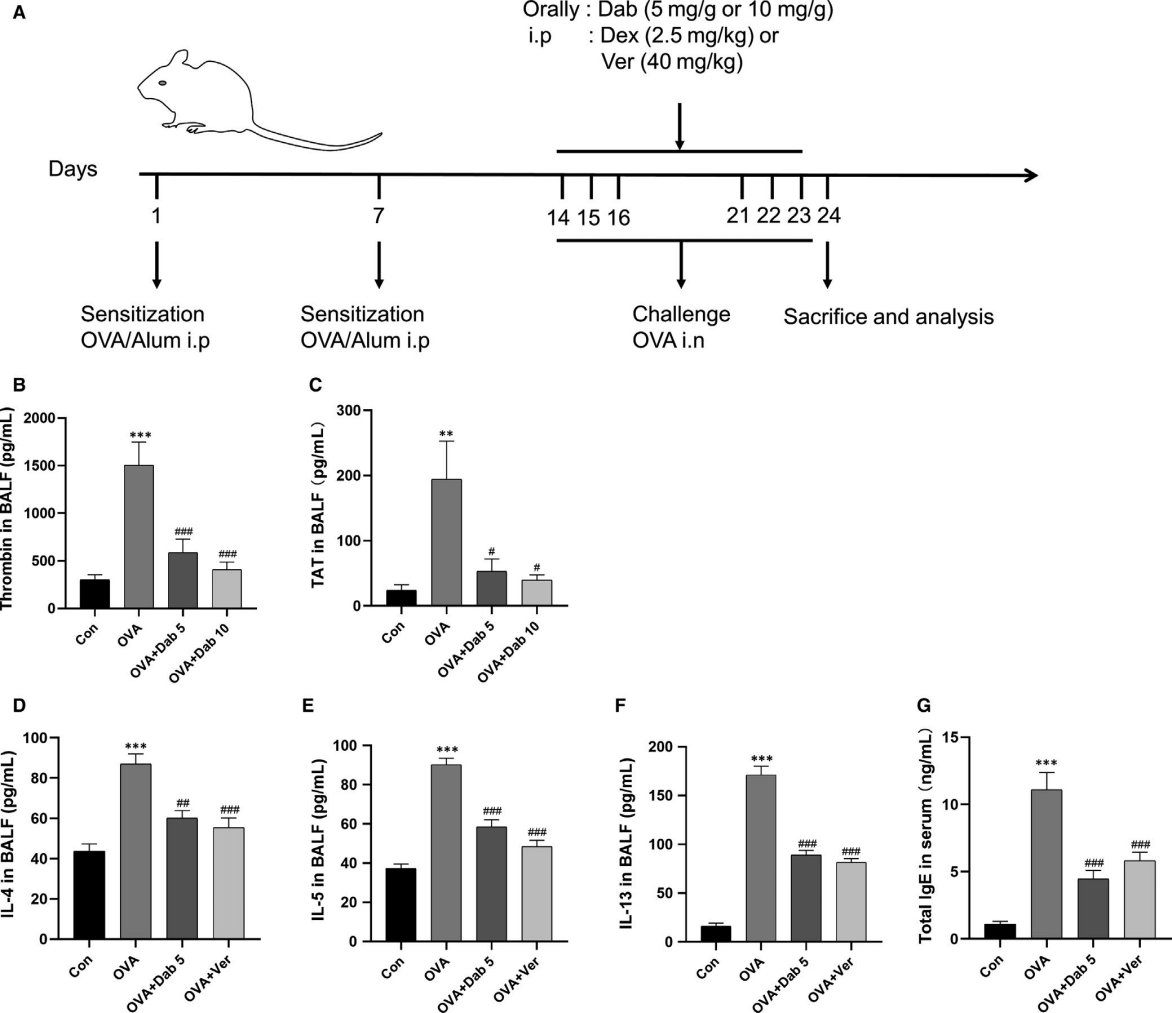
Dabigatran reduced OVA‐induced elevated levels of thrombin and inflammatory cytokines in the airway of mice. A, The construction of OVA‐evoked asthma mouse model. Mice were sensitized by OVA/Al(OH)_3_ on day 1 and day 7, while from day 14 to day 16 and day 21 to 23, mice were challenged with OVA alone by intranasal (i.n.). B‐G, Levels of thrombin, TAT, IL‐4, IL‐5, IL‐13 in BALF and total IgE in serum were measured by ELISA. Data are presented as mean ± SEM. n = 6 mice per group; **P < .01, ***P < .001, compared with the control group; ^#^ *P* < .05, ^##^ *P* < .01, ^###^ *P* < .001, compared with the OVA group

Male BALB/c mice, weighing 25 g, aged 6‐8 weeks, were purchased from Laboratory Animal Center of Southern Medical University, and the animal experimental procedures were approved by the Animal Research Ethics Committee of Southern Medical University. All mice were housed in the SPF facility, and the mice were fed with sterile water and radiation‐irradiated feed for a 12‐hour light/dark cycle. Mice were treated as shown in Figure 1A. Mice were randomly divided into 6 groups according to the experimental requirements (n = 6 for each group): (a) Control group (Con); (b) OVA asthma model group (OVA); (c) OVA + Dabigatran 5 mg/g group (OVA + Dab 5); (d) OVA + Dabigatran 10 mg/g group (OVA + Dab 10); (e) OVA + Verteporfin group (OVA + Ver); and (f) OVA + Dexamethasone group (OVA + Dex). Antigen sensitization was performed by intraperitoneal (i.p.) injection of 50 μg Ovalbumin (Sigma) mixed with 2 mg aluminium hydroxide (Thermo) on day 1 and day 7. On days 14, 15, 16, 21, 22 and 23, mice were challenged with 50 μg OVA alone by intranasal (i.n.). From day 14 to day 23, the dabigatran intervention group began to be fed the corresponding concentration of feed, while the dexamethasone intervention group and verteporfin intervention group were given intraperitoneal injection of the corresponding amount of solution 30 minutes before the challenge of nasal stimulation. Mice were sacrificed within 24 hours after the last challenge, and then, bronchoalveolar lavage fluid and lung tissue were collected.

### 2.3 Lung histology and immunohistochemistry analysis

The lungs were dissected from the chest cavity after the lavage. The right lung was immediately fixed in 4% paraformaldehyde and embedded in paraffin, and tissue sections (5 μm) were prepared. To assess airway remodeling, HE staining was performed to assess airway thickness and inflammatory infiltration, PAS to quantify airway goblet cells and mucus production, and Masson's trichrome to visualize collagen deposition, as described previously.^24^, ^25^, ^26^ Briefly, inflammatory infiltration scores are as follows: 0, no inflammatory cells detected; 1, occasional inflammatory cells; 2, most bronchial or blood vessels only surround a layer of inflammatory cells; 3, most bronchial and perivascular there are 2 layers of inflammation cells; and 4, most of the bronchial or perivascular wrap around more than 2 layers of inflammatory cells. In addition, PAS‐positive cells were expressed per 100 µm of basement membrane and the mean collagen thickness = collagen deposition area/length (μm) of basement membrane.

For immunohistochemical analysis, the sections were initially incubated with anti‐YAP rabbit monoclonal antibody at 4°C overnight and then were incubated with HRP‐conjugated goat anti‐rabbit for 30 minutes at 37°C. Positive staining was detected with HRP‐conjugated streptavidin, visualized with 3, 3′diaminobenzidine. Finally, sections were counterstained with haematoxylin and observed under a light microscope.

### 2.4 Enzyme‐linked immunosorbent assay

The levels of thrombin (Abcam), thrombin‐antithrombin complexes (TAT), interleukin‐4 (IL‐4), IL‐5, IL‐13 in BALF and total serum IgE (Elabscience) were measured by Enzyme‐linked immunosorbent assay (ELISA) kits, respectively. The protocols were followed according to the manufacturer's instructions. Briefly, the standard working solution and the sample to be tested were separately added to a 96‐well plate at 100 μL per well. One hundred microliter of biotin‐conjugated antibody was added to each well and incubated at 37°C for 1 hour. Next, 100 μL of the enzyme conjugate working solution was added per well and incubated at 37°C for 30 minutes. Subsequently, a substrate solution (TMB) was added to each well at 90 μL per well and incubated at 37°C for 15 minutes in the dark. Finally, 50 μL of the stop solution was added to each well to terminate the reaction. The optical density (OD) of each well was measured using a microplate reader set to 450 nm. A standard curve of the average of the OD repeat readings is created to calculate the concentration to be tested.

### 2.5 Culture and stimulation of HASMCs

Human airway smooth muscle cells were isolated from healthy segments of the lobar or main bronchus obtained from three different lung resection donors after giving informed consents, which were approved by the Ethics Committee of Nanfang Hospital, Southern Medical University (NFEC‐201109‐K1). Pure ASM bundles were dissected from surrounding tissues. Cells were maintained as primary culture in DMEM supplemented with 10% FBS, 100 U/mL penicillin and 100 μg/mL streptomycin at 37°C in humidified air containing 5% CO_2_. After 20 to 25 days, HASMCs in primary culture grew to confluence. HASMCs were used between third and sixth passages in all experiments. The characteristics of primary HASMCs are displayed in Supplementary Figure 1 .

Cells were washed twice with phosphate‐buffered saline (PBS) before stimulation and then starved for 12 hours with serum‐free DMEM. Cells were treated with thrombin (1 U/mL) for different lengths of time in different experiments. Different inhibitor groups were treated for 1 hour before treatment with thrombin.

### 2.6 Transfection with small interfering RNA

To knock down the expression of YAP, siRNA (RiboBio) was transfected into HASMCs with Lipofectamine™ 3000 reagent (Invitrogen) according to the manufacturer's instruction. Briefly, Lipofectamine and siRNA were diluted using Opti‐MEM™ I Reduced Serum Medium (Gibco), respectively. Then, the two mixtures were combined and incubated for 15 minutes at room temperature. Finally, the above mixture was added to a cell culture dish and cultured for another 48 hours in a humidified incubator containing 5% CO_2_ at 37°C. The synthetic sequence of siRNA‐YAP is as follows: siRNA‐YAP#1: CCACCAAGCTAGATAAAGA; siRNA‐YAP#2: GAGATGGAATGAACATAGA; siRNA‐YAP#3: GTAGCCAGTTACCAACACT. Control group cells were treated with non‐targeting siRNA (siNC).

### 2.7 RNA extraction and quantitative real‐time PCR

Total RNA was extracted from HASMCs using RNAiso Plus (Takara) in accordance with the manufacture's protocol. The mRNA was quantified by a NanoDrop Spectrophotometer (NanoDrop Tech), and cDNA was synthesized with the PrimeScript™ RT Master Mix (Takara). The gene‐specific primers (listed in Table 1) were obtained from Sangon Biotech. Real‐time quantitative PCR (qPCR) was performed on a LightCycler 480 II (Roche) with a SYBR Premix Ex Taq kit (Takara). The level of GAPDH mRNA expression was used as the internal reference. Data are presented as the fold change over the control group, as determined using the 2^−△△^ *^C^* ^t^ method.

**Table 1:** Primers used for real‐time qPCR assay

Gene	Primer sequence
Collagen I	Forward: 5′‐AGCCAGCAGATCGAGAACAT‐3′
	Reverse: 5′‐TCTTGTCCTTGGGGTTCTTG‐3′
α‐SMA	Forward: 5′‐ ATGCTCCCAGGGCTGTTTTC‐3′
	Reverse: 5′‐ CTTTTGCTCTGTGCTTCGTC‐3′
CTGF	Forward: 5′‐CAGCATGGACGTTCGTCTG‐3′
	Reverse: 5′‐AACCACGGTTTGGTCCTTGG‐3′
Cyclin D1	Forward: 5′‐GCTGCGAAGTGGAAACCATC‐3′
	Reverse: 5′‐CCTCCTTCTGCACACATTTGAA‐3′
PAR1	Forward: 5′‐ CCACCTTAGATCCCCGGTCAT‐3′
	Reverse: 5′‐ GTGGGAGGCTGACTACAAACA‐3′
PAR2	Forward: 5′‐ CAGTGGCACCATCCAAGGAA‐3′
	Reverse: 5′‐ CAGGGCCATGCCGTTACTT‐3′
PAR3	Forward: 5′‐ GCAAAGCCAACCTTACCCATT‐3′
	Reverse: 5′‐ GAGGTAGATGGCAGGTATCAGT‐3′
PAR4	Forward: 5′‐ GCTGCTGCATTACTCGGAC‐3′
	Reverse: 5′‐ ACGTAGGCACCATAGAGGTTG‐3′

### 2.8 Western blot analysis and quantification

Proteins from cultured cells or animal lung tissues were extracted using radioimmunoprecipitation assay (RIPA) lysis buffer (FDbio Science), containing a protease inhibitor cocktail and phosphatase inhibitors (Sigma). Protein concentration was determined by the BCA Protein Assay Kit (FDbio science). For immunoblotting, equal amounts of proteins (20 μg/lane) were subjected to electrophoresis on a 10% or 12% SDS‐polyacrylamide gel and electroblotted to PVDF membrane (EMD Millipore). The membranes were blocked for 1 hour with 5% non‐fat milk in Tris‐Buffered Saline Tween‐20 (TBST). Blots were then incubated over night with specific primary antibodies at 4°C and washed, followed by a 1‐hour incubation with secondary antibodies conjugated to HRP (FDbio science) at room temperature. Target proteins were recorded by using enhanced chemiluminescence (ECL) reagents (FDbio science) and quantified using the ImageJ software.

### 2.9 Immunofluorescence staining

Human airway smooth muscle cells were fixed with 4% paraformaldehyde for 15 minutes and then permeabilized with 0.3% Triton X‐100 for 10 minutes. After blocking for 1 hour in 5% goat serum, cells were incubated with primary antibodies overnight at 4°C and followed by fluorescent‐conjugated anti‐human secondary antibody for 1 hour at room temperature in the next day.

Actin‐Tracker Green (Beyotime Biotech) was used to stain actin filaments, and DAPI (Beyotime Biotech) was used for cell nuclei. Photographs were taken by a fluorescence inverted/laser scanning confocal microscope (Leica Imaging Systems).

### 2.10 Cell proliferation assay

Cell counting kit‐8 (CCK‐8) assay was performed to detect the effects of dabigatran at different concentrations and different treatment times on the proliferation of HASMCs induced by thrombin. HASMCs were seeded in 96‐well plates at a density of 8 × 10^3^ cells/well and incubated overnight at 37°C. After the cells were stimulated with the pre‐designed concentration and time, the CCK‐8 solution was added at 10 μL per well, and then the optical density (OD) value of each well was detected at the wavelength of 450 nm by microplate reader (Spectra Max MD5; Molecular Devices).

EdU (5‐Ethynyl‐2′‐deoxyuridine) incorporation assay was performed to investigate the impact of dabigatran or YAP knockdown on the proliferation of human HASMCs using the Cell‐Light™ EdU imaging detecting kit (RiboBio) according to the manufacturer's instructions. The cells were seeded in six‐well plates, and after treatment, 50 μmol/L EdU solution was added to each well and incubated at 37°C for 2 hours. Subsequently, cells were fixed with 4% paraformaldehyde for 15 minutes and permeabilized with 0.5% Triton X‐100 for 15 minutes. Next, 1 × Apollo^®^ staining reaction solution was added to the cells for 30 minutes in the dark, and then the nuclei were stained with DAPI for 20 minutes. Finally, EdU‐stained cells were observed under an inverted fluorescence microscope (Olympus‐FL 500; Olympus Corporation). The proliferation rate of HASMCs is expressed as the ratio of EdU‐positive cells (red) to total DAPI positive cells (blue).

### 2.11 Scratch migration assay

Human airway smooth muscle cells were seeded into 6‐well plates and grown to a confluent density. After serum starvation and pre‐treatment, the linear wound was scratched in the cell monolayer with a sterile 200 μL pipette tip and then washed three times with PBS to remove cell debris. After the addition of the different treatments, photographs of each wound were taken at different time‐points (0 and 24 hours) in the same field of view to determine the migration of the cells to the wound. All images were captured by an inverted fluorescence microscope (Olympus‐FL 500; Olympus Corporation). The migrated cells were quantified using the ImageJ software program.

### 2.12 Co‐immunoprecipitation

The cells were lysed on ice with Cell Lysis Buffer (Cell Signaling Technology) supplemented with protease inhibitor. Whole cell lysates were incubated with the indicated antibodies overnight at 4°C with gentle rotation, then protein A/G agarose (Millipore) was added and the mixture incubated for 1 hours at room temperature. The immune complexes were collected by centrifugation and washed 3 times with Cell Lysis Buffer, and then the beads‐antibody complex was suspended in the protein lysate. The agarose‐bound protein was eluted by boiling in 3 × SDS‐PAGE loading buffer. Finally, immunoprecipitated protein complexes were detected by Western blotting.

### 2.13 Statistical analysis

All values are presented as mean ± SEM. Differences between two mean values were analysed using Student's *t* test, and multiple mean values were compared using one‐way ANOVA followed by the Tukey post hoc test. Statistical analysis was performed in Prism 8 software (GraphPad). Values of *P* < .05 were considered statistically significant.

## 3 RESULTS

### 3.1 Dabigatran reduced OVA‐induced elevated level of thrombin in the airway of mice

The levels of thrombin and TAT in BALF of mice were determined by ELISA. As shown in Figure 1B,C, compared with the control mice, the levels of thrombin and TAT were greater in the OVA‐evoked asthmatic mice, which were significantly decreased by two different concentrations of dabigatran treatment (5 and 10 mg/g).

### 3.2 Dabigatran or verteporfin attenuated OVA‐induced airway inflammation, remodeling and YAP activation in the lungs of mice

**Figure 2:**
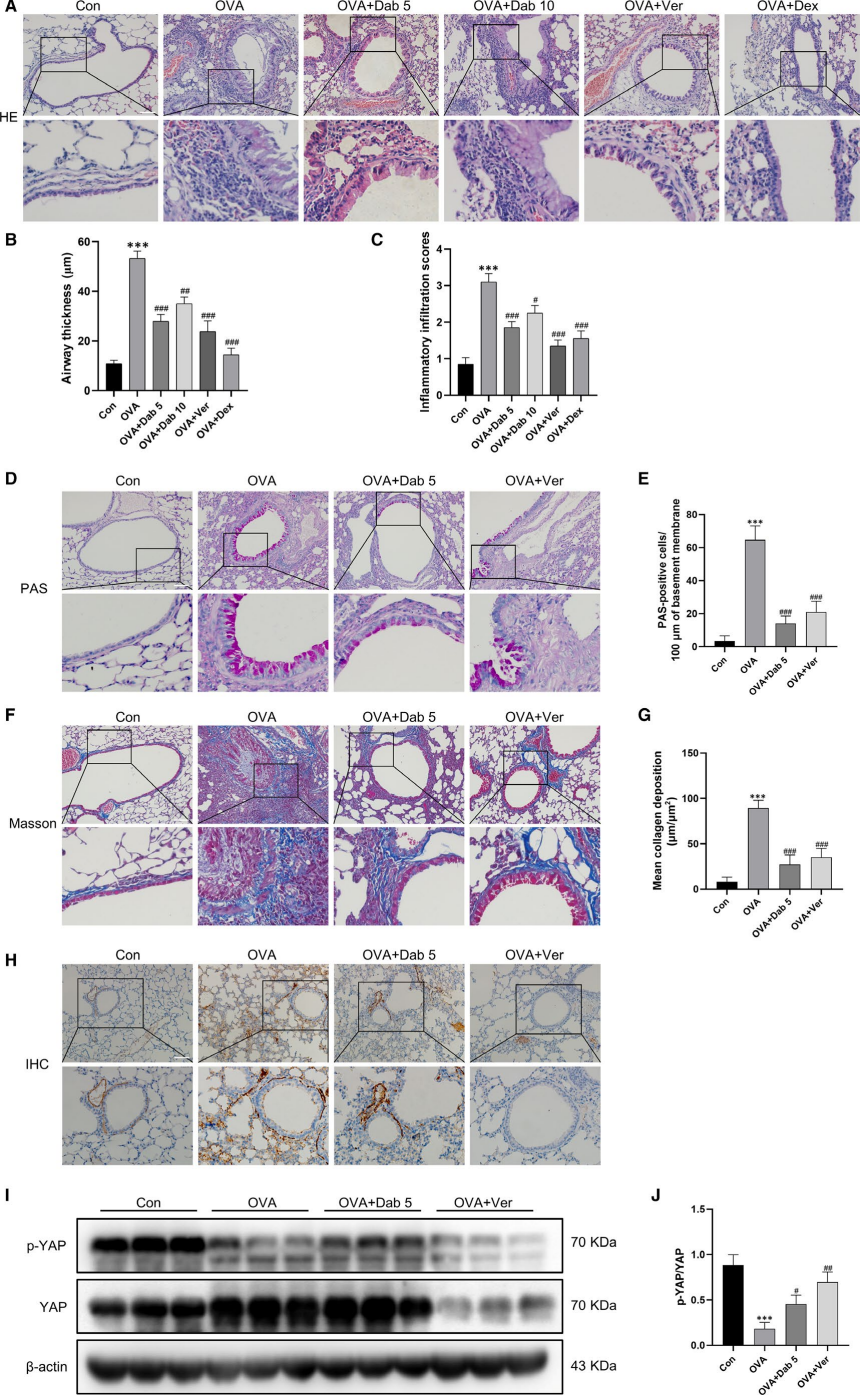
Dabigatran or verteporfin attenuated OVA‐induced lung pathology and YAP activation in mice. A, HE staining was performed to evaluate airway thickness and inflammatory infiltration. D, PAS staining was performed to show goblet cells and mucus production. F, Masson's trichrome staining was performed to determined collagen deposition. H, Immunohistochemical staining for YAP was performed in the lungs. I, Lung tissues from each group were extracted for Western blot to analyse the protein levels of p‐YAP and YAP. (B and C), (E), (G) and (J) are the corresponding quantification histograms of (A), (D), (F) and (I), respectively. Scale bar, 100 μm. Data are presented as mean ± SEM. n = 6 mice per group; ***P < .001, compared with the control group; ^#^ *P* < .05, ^##^ *P* < .01, ^###^ *P* < .001, compared with the OVA group

As shown in Figure 1D‐G, the levels of IL‐4, IL‐5 and IL‐13 in BALF and total IgE in serum were obviously increased in the OVA‐evoked asthmatic mice compared with the control mice, while treatment with dabigatran or verteporfin reduced the levels of IL‐4, IL‐5, IL‐13 and total IgE in comparison with the OVA‐evoked asthmatic mice. Next, HE, PAS and Masson's trichrome staining were conducted. Compared with the control mice, the OVA‐evoked asthmatic mice exhibited thicker airway wall, more inflammatory cell infiltration, goblet cells and mucus production, and collagen deposition. However, these histopathological changes were notably alleviated in the presence of dabigatran and verteporfin compared with the OVA‐evoked asthmatic mice (Figure 2A‐G).

Then, immunohistochemistry and Western blot of mice lung tissue were performed to explore the expression and distribution of YAP and the effect of dabigatran on YAP. Figure 2H depicted that YAP expression was apparently up‐regulated in the OVA‐evoked asthmatic mice and mainly distributed in airway smooth muscle. However, treatment with dabigatran or verteporfin in asthmatic mice reduced YAP expression compared with the OVA‐evoked asthmatic mice. As shown in Figure 2I and J, compared with the control mice, the level of phosphorylated YAP (p‐YAP) was decreased in the OVA‐evoked asthmatic mice. Treatment with dabigatran or verteporfin prevented alterations in the level of p‐YAP induced by OVA challenge.

In summary, the above results indicated that YAP was activated in airway smooth muscle of asthmatic mice, while dabigatran and verteporfin not only inhibited the activation of YAP, but also attenuated the lung pathology of asthma models.

### 3.3 Dabigatran suppressed thrombin‐induced HASMCs remodeling and YAP activation

**Figure 3:**
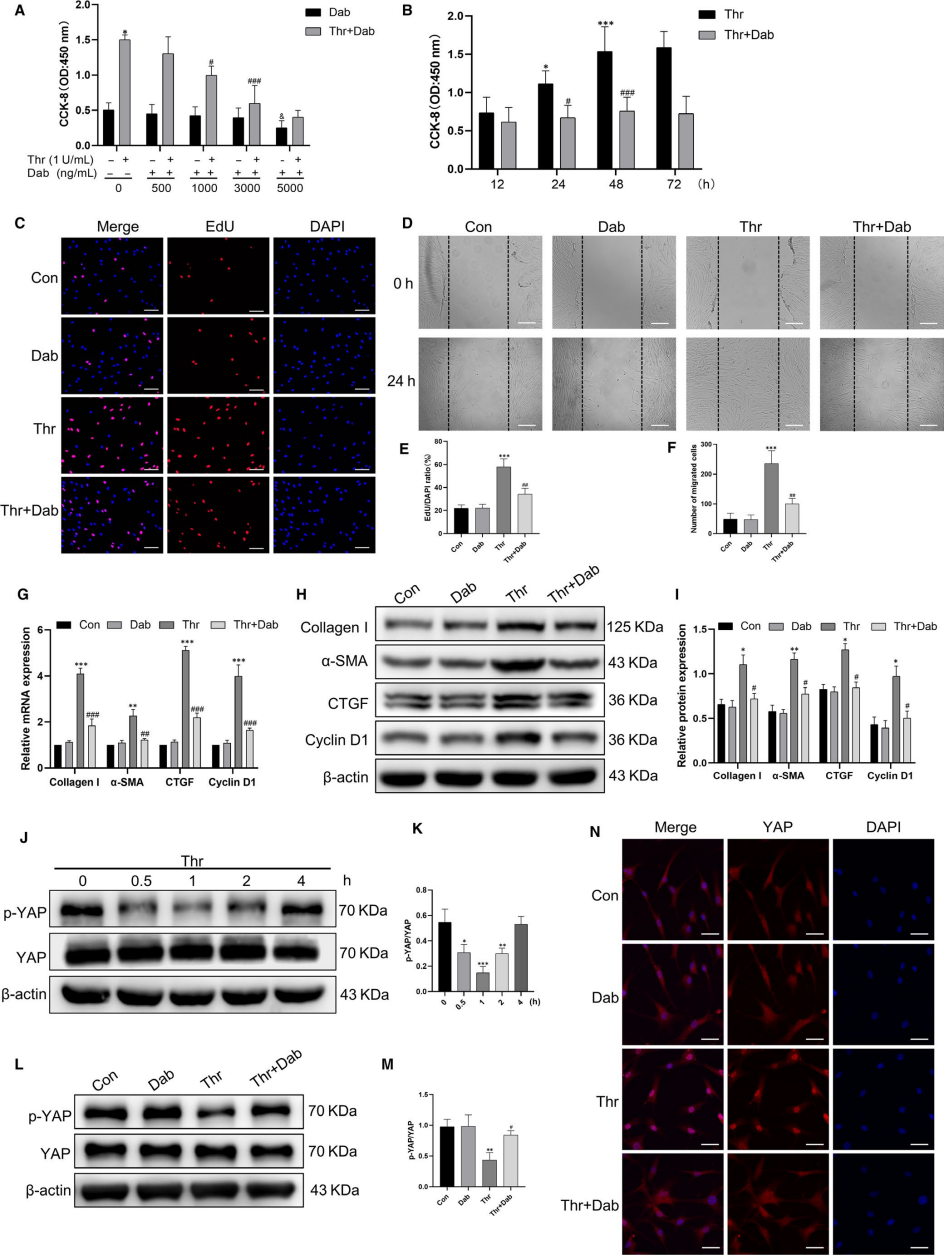
Dabigatran suppressed thrombin‐induced HASMCs remodeling and YAP activation. A, HASMCs were treated with different concentrations of dabigatran (0‐5000 ng/mL) and thrombin (1 U/mL) for 24 h, and cell proliferation was measured by CCK‐8 assay. B, HASMCs were exposed to dabigatran (3000 ng/mL) and thrombin (1 U/mL) for indicated times, and cell proliferation was measured by CCK‐8 assay. C, HASMCs were pre‐treated with dabigatran (3000 ng/mL) for 1 h before stimulation with thrombin (1 U/mL) for 24 h, HASMCs proliferation was measured by EdU incorporation assay. HASMCs were stained with EdU (red) and counterstained with DAPI (blue) to visualize the nuclei (scale bar, 100 μm). D, HASMCs migration was assessed by scratch migration assay (scale bar, 100 μm). G, The mRNA levels of collagen I, α‐SMA, CTGF and cyclin D1 were detected by qPCR. H, The protein levels of collagen I, α‐SMA, CTGF and Cyclin D1 were analysed by Western blot. J, HASMCs were treated with thrombin (1 U/mL) for indicated times, and Western blot was performed to detect the protein levels of p‐YAP and YAP. L, HASMCs were pre‐treated with dabigatran (3000 ng/mL) for 1 h before stimulation with thrombin (1 U/mL) for 1 h, and Western blot was performed to detect the protein levels of p‐YAP and YAP. N, HASMCs were subjected to immunofluorescence staining for YAP (red) to determine its subcellular localization and counterstained with DAPI (blue) to visualize the nuclei under a confocal microscope (scale bar, 50 μm). (E), (F), (I), (K) and (M) are the corresponding quantification histograms of (C), (D), (H), (J) and (L), respectively. All data are presented as mean ± SEM of at least three independent experiments. *P < .05, **P < .01, ***P < .001, compared with the untreated cells; ^#^ *P* < .05, ^##^ *P* < .01, ^###^ *P* < .001, compared with the cells treated with thrombin; ^&^ *P* < .05, compared with the cells treated with dabigatran

We further determined the effects of dabigatran on the cellular biological functions associated with HASMCs remodeling and on YAP activity in response to thrombin. As shown in Figure 3A,B, thrombin effectively stimulated cell proliferation, while pre‐treatment with dabigatran suppressed thrombin‐induced cell proliferation in a concentration‐ and time‐dependent manner, with maximal decrease observed at 3000 ng/mL and 48 hours, respectively. The above results were further validated in the EdU experiment (Figure 3C,E). Moreover, the effects of thrombin and dabigatran on HASMCs migration were tested. The number of migrated cells in the thrombin‐treated group was greater than that in the control group, whereas pre‐treatment with dabigatran significantly impeded thrombin‐induced HASMCs migration (Figure 3D,F). Figure 3G‐I illustrated that thrombin also aggravated HASMCs remodeling by up‐regulating the mRNA and protein expressions of collagen I, α‐SMA, CTGF and cyclin D1, but these alterations induced by thrombin were partially eliminated by pre‐treatment with dabigatran. On the other hand, thrombin resulted in a time‐dependent decrease in the expression of p‐YAP and reached a minimum at 1 hour (Figure 3J,K). However, the presence of dabigatran reversed thrombin‐induced YAP dephosphorylation and nuclear translocation (Figure 3L‐N). These results indicated that dabigatran suppressed thrombin‐induced HASMCs remodeling and YAP activation.

### 3.4 YAP mediated the effects of thrombin on HASMCs remodeling

**Figure 4:**
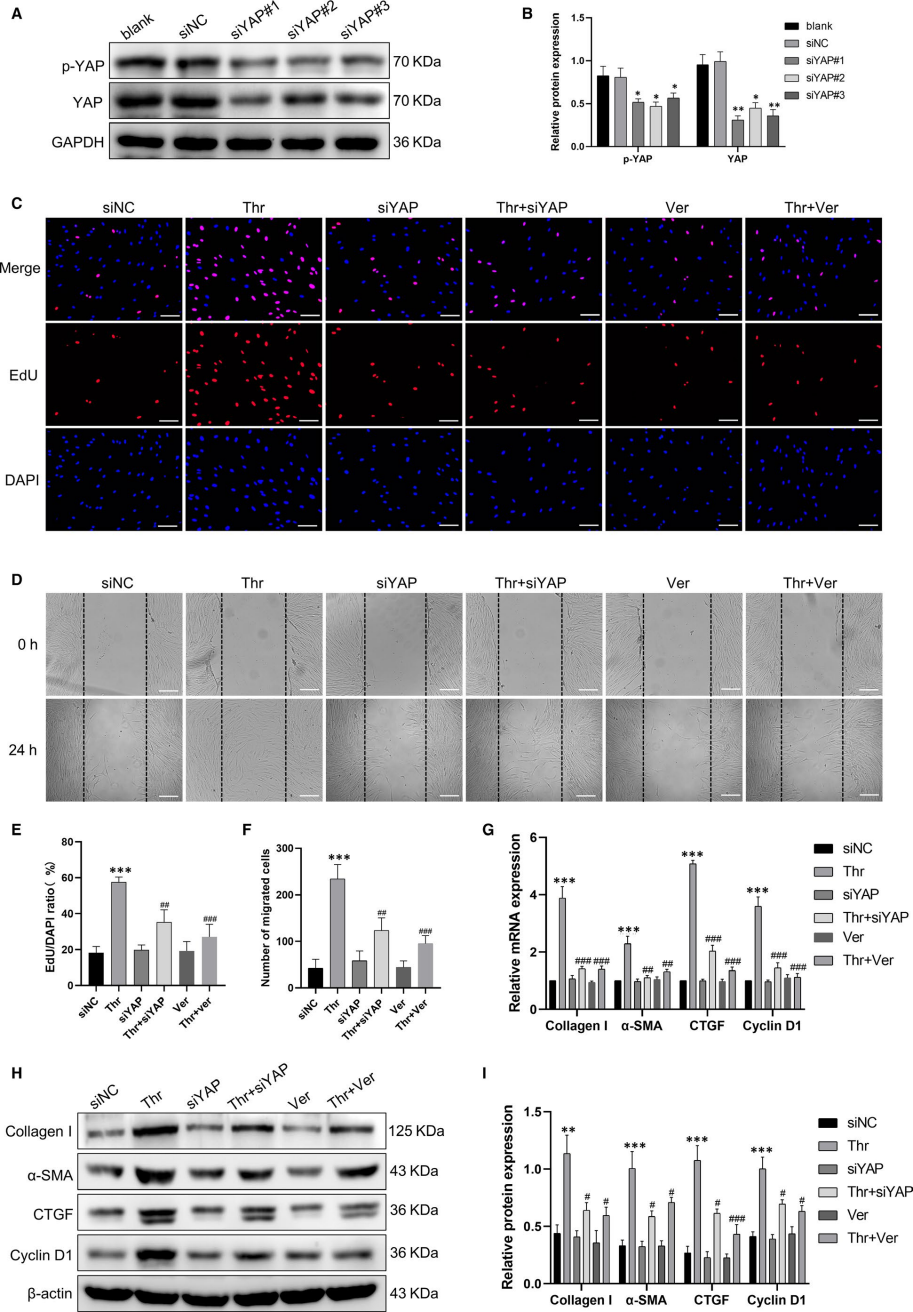
YAP mediated the effects of thrombin on HASMCs remodeling. A, HASMCs were pre‐transfected with YAP siRNA (siYAP) or non‐targeting siRNA (siNC) for 48 h, and the silencing effect was assessed by Western blot. HASMCs were pre‐transfected with siYAP for 48 h or pre‐treated with verteporfin for 1 h and followed by thrombin (1 U/mL) stimulation for 24 h. C, HASMCs proliferation was measured by EdU incorporation assay. HASMCs were stained with EdU (red) and counterstained with DAPI (blue) to visualize the nuclei (scale bar, 100 μm). D, HASMCs migration was assessed by scratch migration assay (scale bar, 100 μm). G, The mRNA levels of collagen I, α‐SMA, CTGF and cyclin D1 were detected by qPCR. H, The protein levels of collagen I, α‐SMA, CTGF and cyclin D1 were analysed by Western blot. (B), (E), (F) and (I) are the corresponding quantification histograms of (A), (C), (D) and (H), respectively. Data are presented as mean ± SEM of at least three independent experiments. *P < .05, **P < .01, ***P < .001, compared with the untreated cells or the cells treated with siNC; ^#^ *P* < .05, ^##^ *P* < .01, ^###^ *P* < .001, compared with the cells treated with thrombin

To investigate the role of YAP in thrombin‐induced proliferation, migration, and up‐regulation of remodeling‐related proteins, siYAP and verteporfin were utilized. The efficiency of YAP knockdown confirmed by immunoblotting, and the best efficiency of three sequences was siYAP#1 (Figure 4A,B). As shown in Figure 4C,E, the proportion of EdU‐positive cells was lower in the thrombin group pre‐transfected with siYAP or pre‐treated with verteporfin than that in the thrombin group. Likewise, both YAP knockdown and verteporfin repressed thrombin‐induced HASMCs migration (Figure 4D,F). Moreover, loss of YAP or YAP inhibition also blocked the up‐regulation of mRNA and protein of collagen I, α‐SMA, CTGF and cyclin D1 induced by thrombin (Figure 4G‐I). These results suggested that YAP activation mediated thrombin‐induced proliferation, migration and up‐regulation of remodeling‐associated proteins in HASMCs.

### 3.5 Thrombin promoted actin stress fibre polymerization through the PAR1/RhoA/ROCK/MLC2 axis to activate YAP

**Figure 5:**
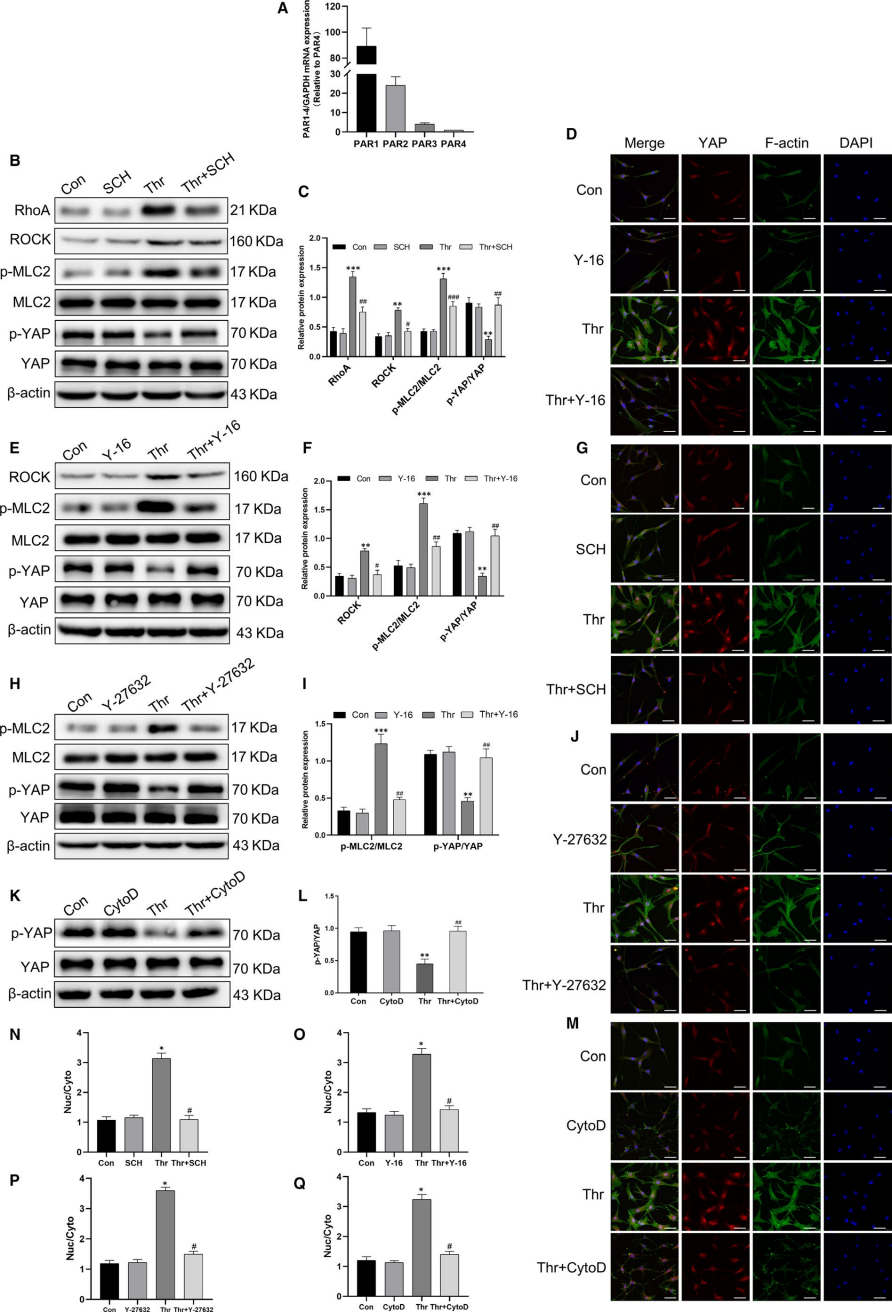
Thrombin promoted actin stress fibre polymerization through the PAR1/RhoA/ROCK/MLC2 axis to activate YAP . A, The mRNA levels of PAR1‐PAR4 were analysed by qPCR. HASMCs were pre‐treated with SCH79797 (10 μmol/L) or Y‐16 (10 μmol/L) or Y‐27632 (10 μmol/L) or Cytochalasin D (10 μmol/L) for 1 h and followed by thrombin (1 U/mL) stimulation for 1 h. B, E, H and K, The protein levels of RhoA, ROCK, p‐MLC2/MLC2 and p‐YAP/YAP were analysed by Western blot. (C), (F), (I) and (L) are the corresponding quantification histograms of (B), (E), (H) and (K), respectively. (D, G, J and M) HASMCs were subjected to immunofluorescence staining for YAP (red) and F‐actin (green), and counterstained with DAPI (blue) to visualize the nuclei under a confocal microscope (scale bar, 50 μm). (N), (O), (P) and (Q) are the corresponding quantification histograms of YAP subcellular localization in (D), (G), (J) and (M), respectively. All data are presented as mean ± SEM of at least three independent experiments. *P < .05, **P < .01, ***P < .001, compared with untreated cells; ^#^ *P* < .05, ^##^ *P* < .01, ^###^ *P* < .001, compared with cells treated with thrombin

The mRNA levels of PAR1‐PAR4 were analysed by qPCR. It was shown that all four PARs were expressed in HASMCs, and PAR1 was the most abundant (Figure 5A). To study the role of PAR1 in thrombin‐induced YAP activation, cells were treated with the PAR1 antagonist SCH79797. Consequently, the presence of SCH79797 substantially prevented thrombin‐induced YAP dephosphorylation and nuclear translocation (Figure 5B,C,D and N). In addition, thrombin up‐regulated the expressions of RhoA, Rho‐associated protein kinase (ROCK), phosphorylated myosin light chain 2 (p‐MLC2) and the polymerization of F‐actin, while SCH79797 suppressed these alterations. Next, pre‐treatment with the RhoA inhibitor Y‐16 significantly blocked thrombin‐induced YAP dephosphorylation and nuclear translocation, as well as up‐regulation of ROCK, p‐MLC2, and polymerization of F‐actin (Figure 5E,F, G and O). Later, ROCK inhibitor Y‐27632 reduced thrombin‐induced YAP dephosphorylation and nuclear translocation, up‐regulation of p‐MLC2, and F‐actin polymerization (Figure 5H,I,J,P). Finally, Cytochalasin D, an inhibitor of actin polymerization, also attenuated thrombin‐induced YAP dephosphorylation and nuclear translocation (Figure 5K,L,M,Q). Based on these observations, we concluded that thrombin induced actin stress fibres by activating PAR1/RhoA/ROCK/MLC2 signalling to regulate YAP dephosphorylation and subcellular localization.

### 3.6 Dabigatran blocked thrombin‐induced interaction between YAP and SMAD2

**Figure 6:**
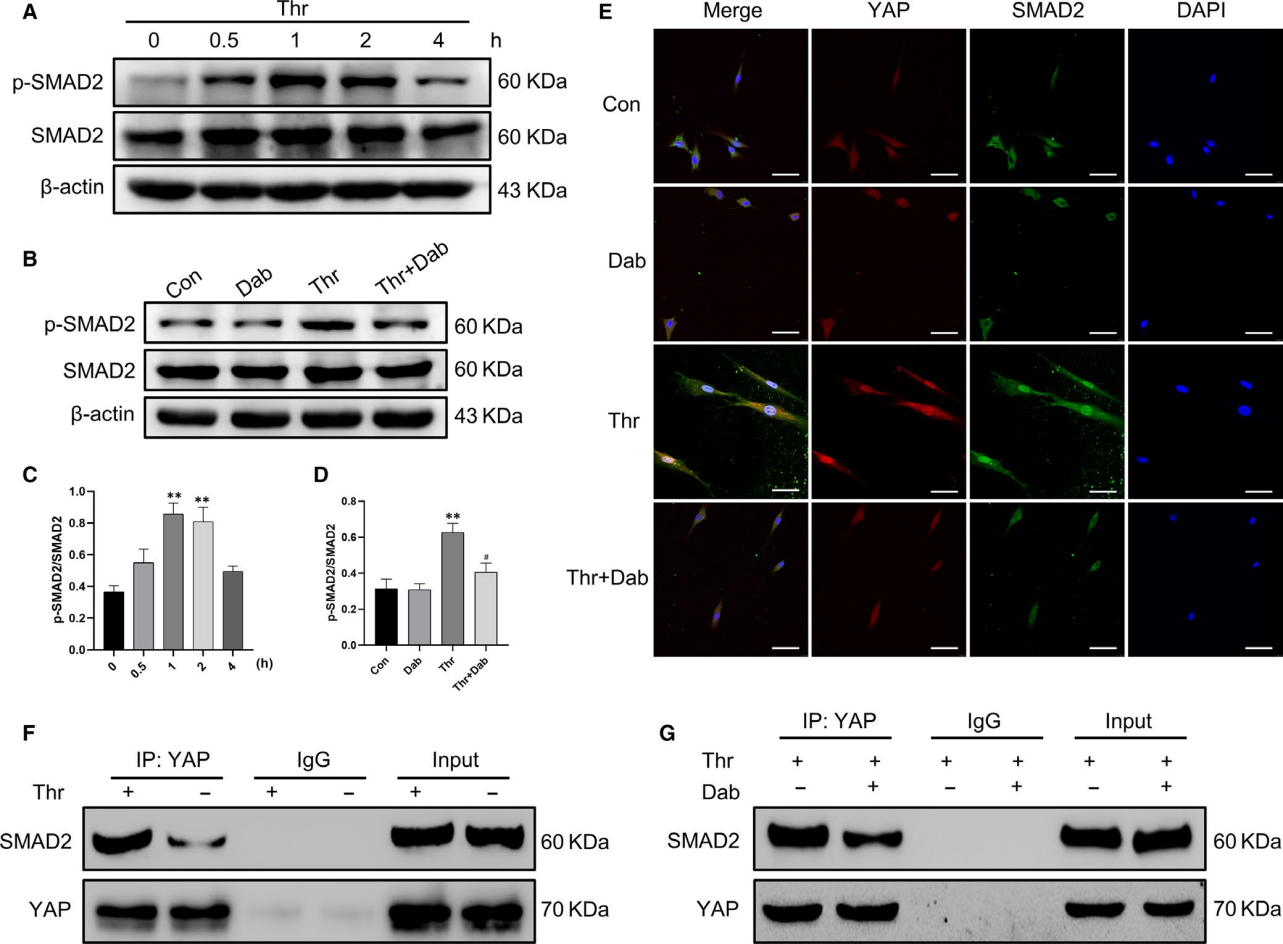
Dabigatran blocked thrombin‐induced interaction between YAP and SMAD2. A, HASMCs were treated with thrombin (1 U/mL) for indicated times, and Western blot was performed to detect the protein levels of p‐SMAD2 and SMAD2. B, HASMCs were pre‐treated with dabigatran (3000 ng/mL) for 1 h before stimulation with thrombin (1 U/mL) for 1 h, and Western blot was performed to detect the protein levels p‐SMAD2 and SMAD2. C and D, are the corresponding quantification histograms of (A) and (B). E, Subcellular localization of YAP and SMAD2 was determined by immunofluorescence. F and G, Co‐immunoprecipitation of YAP with SMAD2. Cell lysates were subjected to immunoprecipitation with YAP antibody or control IgG. The co‐immunoprecipitated SMAD2 was detected by Western blot. Scale bar, 50 μm. All data are presented as mean ± SEM of at least three independent experiments. **P < .01, compared with the untreated cells; ^#^ *P* < .05, compared with cells treated with thrombin

Yes‐associated protein, a transcriptional coactivator, exerts its regulatory functions by binding to transcription factors in the nucleus. We detected that thrombin stimulated SMAD2 phosphorylation in a time‐dependent manner and partially recovered after 1 hour (Figure 6A,C). However, treatment with dabigatran decreased thrombin‐induced SMAD2 phosphorylation (Figure 6B,D). Therefore, we aimed to investigate whether thrombin induced YAP interaction with SMAD2 and whether dabigatran influenced their interaction. YAP and SMAD2 nucleo‐cytoplasmic localization was studied in parallel by immunofluorescence double staining, and we found a significant increase in nuclear accumulation of YAP and SMAD2 after thrombin treatment, but dabigatran reduced thrombin‐induced nuclear accumulation of YAP and SMAD2 (Figure 6E). Furthermore, we performed CoIP to explore the binding of YAP to SMAD2. Figure 6F demonstrated that thrombin promoted the binding of YAP to SMAD2. Nevertheless, the presence of dabigatran reversed thrombin‐induced the binding of YAP to SMAD2 (Figure 6G).

## 4 DISCUSSION

The role of airway smooth muscle cells in airway remodeling is being increasingly recognized, with hyperplasia and hypertrophy of this cell type contributing to wall thickness and airway hyper‐responsiveness and, in addition, airway smooth muscle cells being a source of extracellular matrix, and of growth factors and cytokines that promote inflammation and airway remodeling itself.^27^ Therefore, it is particularly important to elucidate new mechanisms of airway smooth muscle remodeling and find new therapeutic targets.

Recent years, thrombin activation has been shown to have a certain relationship with the pathogenesis of asthma, these studies were mainly focused on allergic inflammation, airway hyper‐responsiveness and fibroblast activation, but there are not many studies on whether thrombin is involved in asthmatic airway smooth muscle remodeling. Interestingly, dabigatran, a direct thrombin inhibitor for clinical anticoagulation, was shown to exert anti‐inflammatory and anti‐fibrotic effects in a mouse model of bleomycin‐induced pulmonary fibrosis,^14^ and the work of de Boer JD et al revealed that dabigatran resulted in a modest reduction of lung pathology in a HDM‐induced asthma model,^28^ which might be due to the HDM model is not characterized by, and too short for the development of, airway remodeling or fibrosis. In this study, we demonstrated that dabigatran exerted a crucial therapeutic effect on airway inflammation and remodeling in the OVA‐evoked asthmatic mice. What's more, dabigatran was reported to reduce thrombin‐induced cell proliferation, α‐SMA expression and tissue, and production of collagen and CTGF in lung fibroblasts.^15^ Besides, Paola Altieri et al described that thrombin stimulated the proliferation and contraction of primary human atrial fibroblasts and the expression of α‐SMA, collagen I and fibronectin, all of which were prevented by dabigatran.^29^ Here, we found that up‐regulation of HASMCs proliferation, migration and the expression of collagen I, α‐SMA, CTGF and cyclin D1 induced by thrombin were notably impaired by dabigatran. Taking together, we demonstrated that dabigatran relieved airway smooth muscle remodeling in vivo and in vitro.

The hippo pathway is an evolutionarily conserved signalling cascade that controls organ size by regulating cell proliferation, apoptosis, differentiation and stem/progenitor cell fate, which involves a series of kinases and adaptors that leads to inactivation of transcriptional coactivator with YAP.^30^ Increased YAP expression has been detected in airway smooth muscle cells and bronchial epithelial cells, suggesting that YAP may participate in the pathogenesis of asthma.^18^, ^31^, ^32^, ^33^ Our experiments confirmed that both YAP knockdown and YAP inhibition were effective in alleviating airway remodeling in asthmatic mice and thrombin‐induced proliferation, migration, and up‐regulation of remodeling‐related proteins in HASMCs. However, the upstream and downstream mechanisms that regulate YAP activity in specific cells and circumstances have not been fully elucidated. GPCRs are the largest family of membrane receptors in mammals, which has been shown to act as an upstream regulatory signal for the hippo/YAP pathway. Specifically, ligand signalling through GPCRs coupled to Gα_12/13_, Gα_i/o_ or Gα_q/11_, such as LPA, angiotensin II and oestrogen, activates YAP; in contrast, ligand signalling through GPCRs coupled to Gαs, such as glucagon and epinephrine, suppresses YAP activity.^34^, ^35^, ^36^ In addition, dynamic modulation of the F‐actin cytoskeleton is required for hippo pathway regulation by GPCR ligands,^37^, ^38^ and it was reported that a key factor in stress fibre formation was Rho‐GTPase‐mediated phosphorylation of MLC2.^39^ It is worth mentioning that Jung‐Soon Mo et al confirmed that PAR1, a receptor for thrombin, regulated YAP dephosphorylation by Gα_12/13_ in MDA‐MB‐231 cells.^20^ In this study, we first confirmed that thrombin induced YAP activation in HASMCs. Afterwards, PAR1 was identified to be the most abundant of the four PARs in HASMCs, and the inhibition of PAR1 suppressed thrombin‐induced YAP activation, suggesting that thrombin activated YAP via PAR1. At the same time, inhibition of PAR1 also reduced thrombin‐induced up‐regulation of RhoA, ROCK, phosphorylated MLC2 and F‐actin polymerization. Likewise, RhoA inhibitor, ROCK inhibitor and F‐actin cytoskeleton inhibitor were applied separately, and thrombin‐mediated YAP activation was significantly inhibited. It is intriguing that YAP activity is regulated by the conformation of the F‐actin cytoskeleton, which in turn primarily depends on the substrate adhesion and intercellular tension,^40^ so the mechanism of mechanical stress signal in thrombin‐induced YAP activation is worthy of further investigation in future studies.

As a transcriptional coactivator, YAP does not contain any intrinsic DNA‐binding domain and exert regulatory functions by binding to transcription factors in the nucleus. Xaralabos Varelas et al^41^ demonstrated that low cell density drove the binding and nuclear accumulation of YAP and SMAD2/3. Interestingly, we detected that thrombin also induced rapid phosphorylation of SMAD2. Therefore, immunofluorescence was performed to detect colocalization of YAP with SMAD2, and CoIP to further verify their interaction. Our results demonstrated that YAP and SMAD2 binded to each other and traslocated to the nucleus after thrombin stimulation, while dabigatran reversed thrombin‐induced these changes.

**Figure 7:**
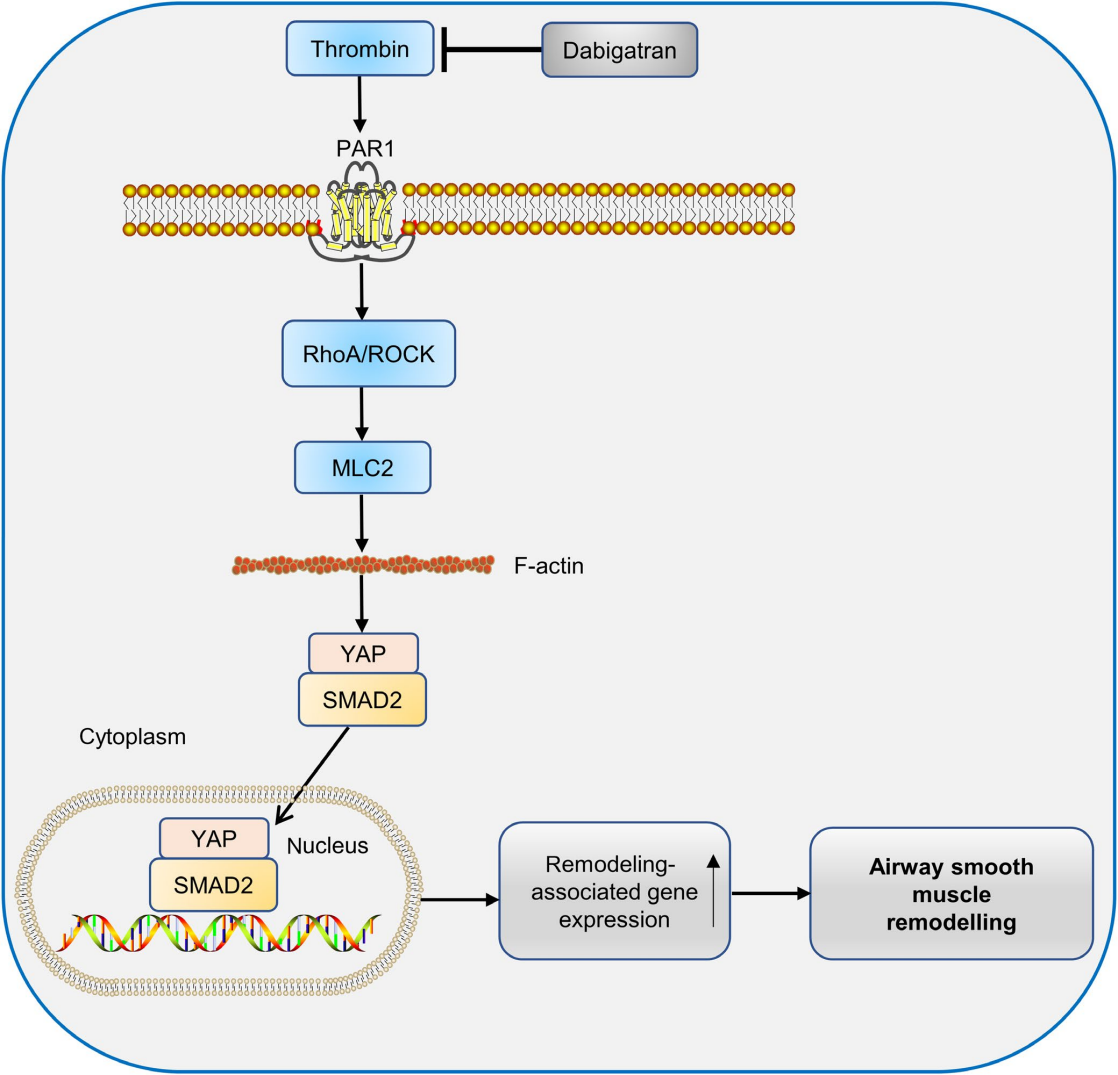
Proposed therapeutic mechanism of dabigatran in alleviating asthmatic airway smooth muscle remodeling

In conclusion, this study is the first to demonstrate that dabigatran reverses airway smooth muscle remodeling effectively in vivo and in vitro. Mechanistically, the protective effect of dabigatran might be associated with the suppression of thrombin‐induced YAP activation. The proposed scheme is displayed in Figure 7.

Currently, the use of anti‐inflammatory drugs and bronchodilators is the principle of conventional drug therapy for asthma. Although these drugs alleviate the symptoms, they do not reverse the established changes in airway structure, especially airway remodeling. Our study may provide some evidence that dabigatran has the potential to relieve airway smooth muscle remodeling in asthmatic patients.

## CONFLICT OF INTEREST

The authors declare no conflicts of interest.

## AUTHOR CONTRIBUTIONS

Yuanxiong Cheng was responsible for the study hypothesis and design. Zhenan Deng carried out the experiments, performed the statistical analysis and drafted the manuscript. Haojun Xie participated in the research design, reviewed and edited the manuscript. Weiying Cheng, Meihong Zhang and Jie Liu helped to conduct the experiments. Yating Huo and Yulin Liao provided experimental facilities.

## Supporting information

Figure S1

## Data Availability

The data that support the findings of this study are available from the first author upon reasonable request.

## References

[1] Papi A , Brightling C , Pedersen SE , Reddel HK . Asthma. Lancet. 2018;391:783‐800. 29273246 10.1016/S0140-6736(17)33311-1

[2] Busse WW , Lemanske RF Jr . Asthma. N Engl J Med. 2001;344:350‐362. 11172168 10.1056/NEJM200102013440507

[3] Cohn L , Elias JA , Chupp GL . Asthma: mechanisms of disease persistence and progression. Annu Rev Immunol. 2004;22:789‐815. 15032597 10.1146/annurev.immunol.22.012703.104716

[4] Jeffery PK . Remodeling in asthma and chronic obstructive lung disease. Am J Respir Crit Care Med. 2001;164:S28‐S38. 11734464 10.1164/ajrccm.164.supplement_2.2106061

[5] Ozier A , Allard B , Bara I , et al. The pivotal role of airway smooth muscle in asthma pathophysiology. J Allergy. 2011;2011:742710. 10.1155/2011/742710 PMC3246780 22220184

[6] de Boer JD , Majoor CJ , van 't Veer C , Bel EHD , van der Poll T . Asthma and coagulation. Blood. 2012;119:3236‐3244. 22262775 10.1182/blood-2011-11-391532

[7] Sneeboer MM , Fens N , van de Pol MA , et al. Loss of asthma control and activation of coagulation and fibrinolysis. Clin Exp Allergy. 2016;46:422‐427. 26509255 10.1111/cea.12667

[8] Gabazza EC , Taguchi O , Tamaki S , et al. Thrombin in the airways of asthmatic patients. Lung. 1999;177:253‐262. 10384063 10.1007/pl00007645

[9] Kanazawa H , Yoshikawa T . Up‐regulation of thrombin activity induced by vascular endothelial growth factor in asthmatic airways. Chest. 2007;132:1169‐1174. 17934112 10.1378/chest.07-0945

[10] Reed CE , Kita H . The role of protease activation of inflammation in allergic respiratory diseases. J Allergy Clin Immunol. 2004;114:997‐1008; quiz 9. 15536399 10.1016/j.jaci.2004.07.060

[11] Hauck RW , Schulz C , Schomig A , Hoffman RK , Panettieri RA Jr . Alpha‐Thrombin stimulates contraction of human bronchial rings by activation of protease‐activated receptors. Am J Physiol. 1999;277:L22‐L29. 10409227 10.1152/ajplung.1999.277.1.L22

[12] Ortiz‐Stern A , Deng X , Smoktunowicz N , Mercer PF , Chambers RC . PAR‐1‐dependent and PAR‐independent pro‐inflammatory signaling in human lung fibroblasts exposed to thrombin. J Cell Physiol. 2012;227:3575‐3584. 22278285 10.1002/jcp.24061

[13] Yao ZH , Xie HJ , Yuan YL , et al. Contraction‐dependent TGF‐beta1 activation is required for thrombin‐induced remodeling in human airway smooth muscle cells. Life Sci. 2018;197:130‐139. 29428600 10.1016/j.lfs.2018.02.012

[14] Bogatkevich GS , Ludwicka‐Bradley A , Nietert PJ , Akter T , van Ryn J , Silver RM . Antiinflammatory and antifibrotic effects of the oral direct thrombin inhibitor dabigatran etexilate in a murine model of interstitial lung disease. Arthritis Rheum. 2011;63:1416‐1425. 21312187 10.1002/art.30255 PMC3086970

[15] Bogatkevich GS , Ludwicka‐Bradley A , Silver RM . Dabigatran, a direct thrombin inhibitor, demonstrates antifibrotic effects on lung fibroblasts. Arthritis Rheum. 2009;60:3455‐3464. 19877031 10.1002/art.24935 PMC2837365

[16] Piccolo S , Dupont S , Cordenonsi M . The biology of YAP/TAZ: hippo signaling and beyond. Physiol Rev. 2014;94:1287‐1312. 25287865 10.1152/physrev.00005.2014

[17] Yu FX , Guan KL . The Hippo pathway: regulators and regulations. Genes Dev. 2013;27:355‐371. 23431053 10.1101/gad.210773.112 PMC3589553

[18] Zhou J , Xu F , Yu JJ , Zhang W . YAP is up‐regulated in the bronchial airway smooth muscle of the chronic asthma mouse model. Int J Clin Exp Pathol. 2015;8:11132‐11139. 26617833 PMC4637648

[19] Liu L , Zhai C , Pan Y , et al. Sphingosine‐1‐phosphate induces airway smooth muscle cell proliferation, migration, and contraction by modulating Hippo signaling effector YAP. Am J Physiol Lung Cell Mol Physiol. 2018;315:L609‐L621. 29999407 10.1152/ajplung.00554.2017

[20] Mo JS , Yu FX , Gong R , Brown JH , Guan KL . Regulation of the Hippo‐YAP pathway by protease‐activated receptors (PARs). Genes Dev. 2012;26:2138‐2143. 22972936 10.1101/gad.197582.112 PMC3465735

[21] Ma L , Dorling A . The roles of thrombin and protease‐activated receptors in inflammation. Semin Immunopathol. 2012;34:63‐72. 21809138 10.1007/s00281-011-0281-9

[22] Aragona M , Panciera T , Manfrin A , et al. A mechanical checkpoint controls multicellular growth through YAP/TAZ regulation by actin‐processing factors. Cell. 2013;154:1047‐1059. 23954413 10.1016/j.cell.2013.07.042

[23] Sun S , Irvine KD . Cellular organization and cytoskeletal regulation of the Hippo signaling network. Trends Cell Biol. 2016;26:694‐704. 27268910 10.1016/j.tcb.2016.05.003 PMC4993636

[24] Huang WC , Fang LW , Liou CJ . Phloretin attenuates allergic airway inflammation and oxidative stress in asthmatic mice. Front Immunol. 2017;8:134. 28243240 10.3389/fimmu.2017.00134 PMC5303714

[25] Li N , He Y , Yang G , Yu Q , Li M . Role of TRPC1 channels in pressure‐mediated activation of airway remodeling. Respir Res. 2019;20:91. 31092255 10.1186/s12931-019-1050-x PMC6518742

[26] Pu Y , Liu YQ , Zhou Y , et al. Dual role of RACK1 in airway epithelial mesenchymal transition and apoptosis. J Cell Mol Med. 2020;24:3656‐3668. 32064783 10.1111/jcmm.15061 PMC7131927

[27] Prakash YS , Halayko AJ , Gosens R , Panettieri RA Jr , Camoretti‐Mercado B , Penn RB . An official American Thoracic Society Research statement: current challenges facing research and therapeutic advances in airway remodeling. Am J Respir Crit Care Med. 2017;195:e4‐e19. 28084822 10.1164/rccm.201611-2248ST

[28] de Boer JD , Berkhout LC , de Stoppelaar SF , et al. Effect of the oral thrombin inhibitor dabigatran on allergic lung inflammation induced by repeated house dust mite administration in mice. Am J Physiol Lung Cell Mol Physiol. 2015;309:L768‐L775. 26320153 10.1152/ajplung.00102.2015

[29] Altieri P , Bertolotto M , Fabbi P , et al. Thrombin induces protease‐activated receptor 1 signaling and activation of human atrial fibroblasts and dabigatran prevents these effects. Int J Cardiol. 2018;271:219‐227. 29801760 10.1016/j.ijcard.2018.05.033

[30] Ma S , Meng Z , Chen R , Guan KL . The Hippo pathway: biology and pathophysiology. Annu Rev Biochem. 2019;88:577‐604. 30566373 10.1146/annurev-biochem-013118-111829

[31] Fu J , Zheng M , Zhang X , et al. Fibulin‐5 promotes airway smooth muscle cell proliferation and migration via modulating Hippo‐YAP/TAZ pathway. Biochem Biophys Res Comm. 2017;493:985‐991. 28942149 10.1016/j.bbrc.2017.09.105

[32] Fodor LE , Gezsi A , Ungvari L , et al. Investigation of the possible role of the Hippo/YAP1 pathway in asthma and allergy. Allergy, Asthma Immunol Res. 2017;9:247‐256. 28293931 10.4168/aair.2017.9.3.247 PMC5352576

[33] Volckaert T , Yuan T , Chao CM , et al. Fgf10‐Hippo epithelial‐mesenchymal crosstalk maintains and recruits lung basal stem cells. Dev Cell. 2017;43(1):48‐59.e5. 29017029 10.1016/j.devcel.2017.09.003 PMC5679744

[34] Yu FX , Zhao B , Panupinthu N , et al. Regulation of the Hippo‐YAP pathway by G‐protein‐coupled receptor signaling. Cell. 2012;150:780‐791. 22863277 10.1016/j.cell.2012.06.037 PMC3433174

[35] Zhou X , Wang S , Wang Z , et al. Estrogen regulates Hippo signaling via GPER in breast cancer. J Clin Investig. 2015;125:2123‐2135. 25893606 10.1172/JCI79573 PMC4463207

[36] Yu FX , Luo J , Mo JS , et al. Mutant Gq/11 promote uveal melanoma tumorigenesis by activating YAP. Cancer Cell. 2014;25:822‐830. 24882516 10.1016/j.ccr.2014.04.017 PMC4075337

[37] Zhao B , Li L , Wang L , Wang CY , Yu J , Guan KL . Cell detachment activates the Hippo pathway via cytoskeleton reorganization to induce anoikis. Genes Dev. 2012;26:54‐68. 22215811 10.1101/gad.173435.111 PMC3258966

[38] Feng X , Degese MS , Iglesias‐Bartolome R , et al. Hippo‐independent activation of YAP by the GNAQ uveal melanoma oncogene through a trio‐regulated rho GTPase signaling circuitry. Cancer Cell. 2014;25:831‐845. 24882515 10.1016/j.ccr.2014.04.016 PMC4074519

[39] Katoh K , Kano Y , Noda Y . Rho‐associated kinase‐dependent contraction of stress fibres and the organization of focal adhesions. J R Soc Interface. 2011;8:305‐311. 20826475 10.1098/rsif.2010.0419 PMC3030825

[40] Panciera T , Azzolin L , Cordenonsi M , Piccolo S . Mechanobiology of YAP and TAZ in physiology and disease. Nat Rev Mol Cell Biol. 2017;18:758‐770. 28951564 10.1038/nrm.2017.87 PMC6192510

[41] Varelas X , Samavarchi‐Tehrani P , Narimatsu M , et al. The Crumbs complex couples cell density sensing to Hippo‐dependent control of the TGF‐beta‐SMAD pathway. Dev Cell. 2010;19:831‐844. 21145499 10.1016/j.devcel.2010.11.012

