# Supplementary figures and images for: Dabigatran ameliorates airway smooth muscle remodeling in asthma by modulating Yes‐associated protein
Source: J Cell Mol Med. 2020 Jun 15;24(14):8179–93. doi: 10.1111/jcmm.15485 (PMC7348141; doi:10.1111/jcmm.15485)

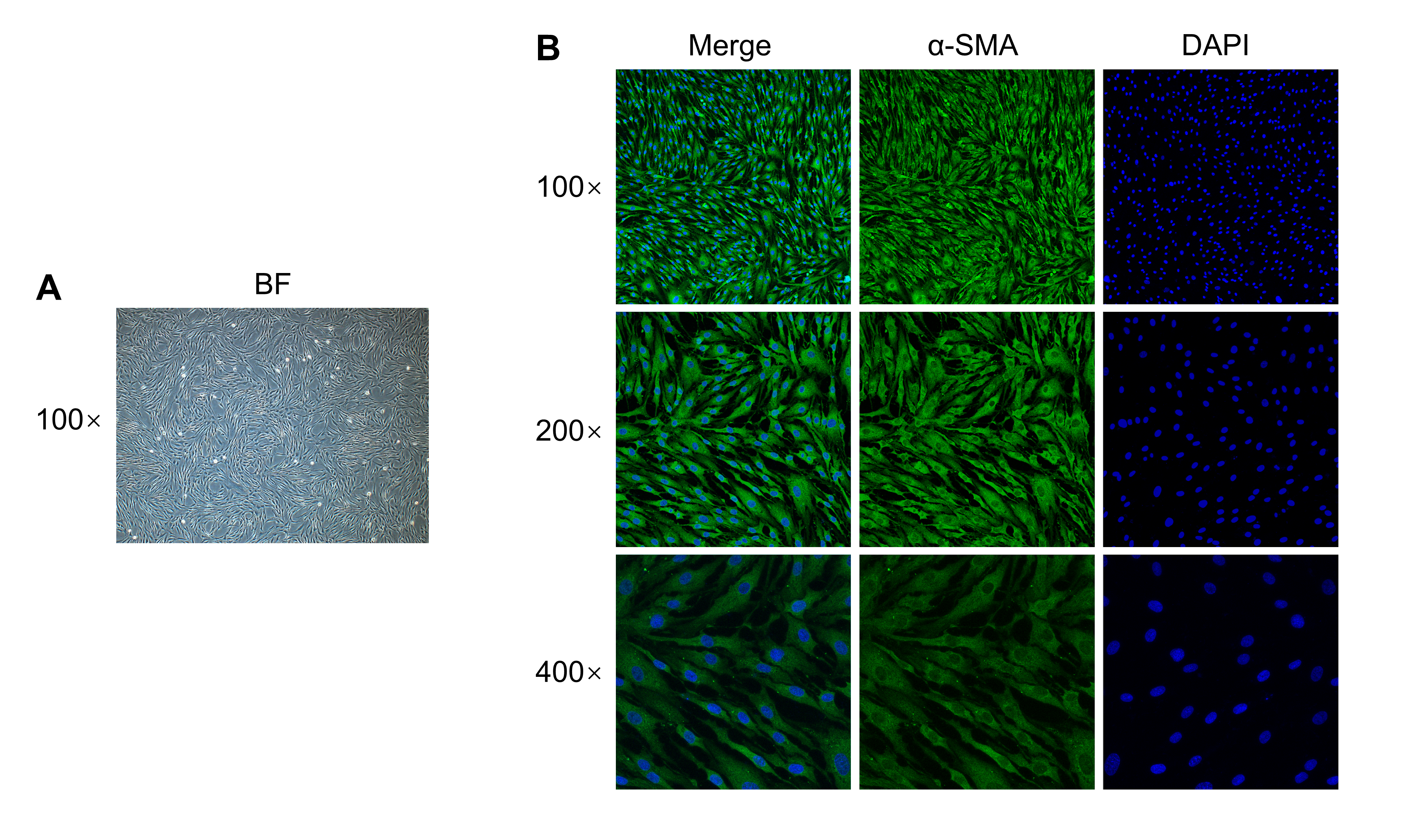

Supplement: Supplementary file 1 — Figure S1 [file JCMM-24-8179-s001.tif]
